# Statin Use Decreases the Risk of Metachronous Gastric Cancer in Patients without *Helicobacter pylori* Infection

**DOI:** 10.3390/cancers13051020

**Published:** 2021-03-01

**Authors:** Tae Jin Kwon, Tae Jun Kim, Hyuk Lee, Yang Won Min, Byung-Hoon Min, Jun Haeng Lee, Jae J. Kim

**Affiliations:** Department of Medicine, Samsung Medical Center, Sungkyunkwan University School of Medicine, 81 Irwon-ro, Gangnam-gu, Seoul 06351, Korea; tj6647.kwon@samsung.com (T.J.K.); tj23.kim@samsung.com (T.J.K.); yangwon.min@samsung.com (Y.W.M.); jason.min@samsung.com (B.-H.M.); jh2145.lee@samsung.com (J.H.L.); jaej.kim@samsung.com (J.J.K.)

**Keywords:** gastric cancer, ESD, statin, prevention

## Abstract

**Simple Summary:**

Statins have been shown to reduce the risk of gastric cancer (GC). Little is known about their effects on metachronous GC in *H. pylori* negative patients after endoscopic resection for early gastric cancer (EGC). In this cohort study among patients with EGC without *H. pylori* infection, we found that statins are associated with a decrease in the risk of recurrence of GC. There was a dose-response relationship between the use of statins and the risk of metachronous GC. This study provides evidence about the additional benefits of statins as chemopreventive agents against metachronous GC among patients who underwent endoscopic resection for EGC.

**Abstract:**

Previous studies have shown that statins reduce the risk of gastric cancer; however, their role has not been adequately studied in patients without *Helicobacter* *pylori* infection. We aimed to investigate whether statins reduced the risk of metachronous gastric cancer (GC) in *H. pylori*-negative patients who underwent endoscopic resection for early gastric cancer (EGC). Retrospective data of 2153 patients recruited between January 2007 and December 2016, with no *H. pylori* infection at baseline, who underwent resection for EGC, were analyzed. Metachronous GC was defined as a newly developed GC at least 1 year after endoscopic resection. Patients who used statins for at least 28 days during the follow-up period were considered as statin users. During a median follow-up of 5 years (interquartile range, 3.5–6.2), metachronous GC developed in 165 (7.6%) patients. In the multivariate Cox regression analysis, statin use was an independent factor associated with GC recurrence (adjusted hazard ratio (HR), 0.46; 95% confidence interval (CI), 0.26–0.82). Moreover, the risk of GC reduced with increasing duration (<3 years: HR 0.40, 95% CI 0.14–1.13; ≥3 years: HR 0.21, 95% CI 0.05–0.90; *p* trend = 0.011) and the dose of statin (cumulative defined daily dose (cDDD) < 500: HR 0.45, 95% CI 0.16–1.28; cDDD ≥ 500: HR 0.19, 95% CI 0.04–0.80; *p* trend = 0.008) in the propensity score-matched cohort. Statin use was associated with a lower risk of GC recurrence in *H. pylori*-negative patients with resected EGC in a dose-response relationship.

## 1. Introduction

Gastric cancer (GC) has the fifth highest incidence and is the third highest cause of cancer-related death globally [1,2]. Early gastric cancer (EGC) is defined as invasive GC confined of the mucosa and submucosa, irrespective of lymph node metastasis. Endoscopic resection as a minimally invasive treatment is indicated for EGC with a negligible risk of lymph node metastasis. However, metachronous GC, defined as a new GC located distant from the site of index GC after 1 year of index endoscopic resection, can develop due to the preservation of the stomach. Known risk factors for metachronous GC include *Helicobacter pylori* infection, age, male sex, and severe gastric mucosal atrophy [3,4]. *H. pylori* infection is the primary cause of gastric adenocarcinoma [5,6]. A recent meta-analysis reported that *H. pylori* eradication was associated with a 47% reduction in the risk of GC [7]. *H. pylori* eradication is also beneficial after the treatment of EGC [8]. In a recent randomized clinical trial of patients who underwent endoscopic resection for EGC, *H. pylori* eradication reduced the risk of metachronous GC by 50% [9]. Since *H. pylori* eradication reduces the risk of metachronous GC by only about 50%, there remains an unmet need to identify effective chemopreventive agents against GC.

Statins are widely used as cholesterol-lowering agents and are known to be competitive inhibitors of β-Hydroxy β-methylglutaryl-coenzyme A (HMG-CoA) reductase. Statins have been found to be associated with a decreased risk of various malignancies in numerous epidemiologic studies [10,11]. In addition to their cholesterol-lowering properties, statins also have antiproliferative and proapoptotic effects [12]. Although there are conflicting results about the benefit of statins in GC, in a case-control study of patients with diabetes from South Korea, statin use was associated with an 80% reduction in the likelihood of developing GC [13]. In contrast, a Dutch study with a pharmacy database found no significant association between statin use and GC [14]. Without stratifying the patients based on their *H. pylori* infection status, the true impact of statin use on the risk of GC cannot be conclusively analyzed. The chemopreventive effect of statins should be investigated in patients with no *H. pylori* infection to eliminate the effect of *H. pylori* infection as a confounding factor. In addition, it is necessary to confirm the effect of statins on the prevention of GC in high-risk patients.

To date, there are no studies that have investigated the chemopreventive effect of statins on metachronous GC in *H. pylori*-negative patients who have undergone resection for EGC. Therefore, we aimed to investigate the chemopreventive effect of statins on metachronous GC in a cohort of *H. pylori*-negative patients who underwent endoscopic resection for EGC.

## 2. Results

### 2.1. Baseline Characteristics of the Study Cohort

In this cohort, 228 patients were statin users, and 1925 patients were statin nonusers. Baseline characteristics of the subjects in both groups are summarized in Table 1. The median age of the study patients was 64 years [interquartile range (IQR), 57–71], and the total cohort of 2153 patients comprised 1699 (78.9%) men and 454 (21.1%) women. Statin users were more likely to take aspirin or metformin and had a higher proportion of patients with dyslipidemia, hypertension, diabetes mellitus, myocardial infarction, heart failure, chronic kidney disease, and cerebrovascular accident. However, there was no difference between statin users and nonusers in terms of age, sex, and tumor characteristics.

### 2.2. Factors Associated with Metachronous GC

**Figure 1:**
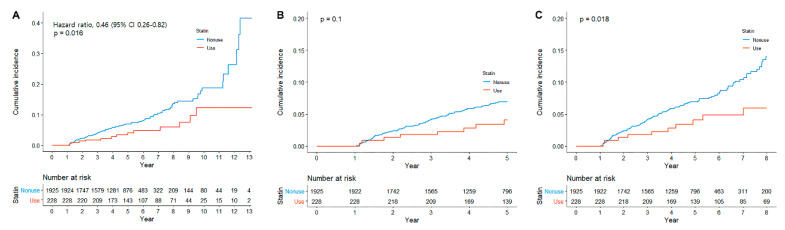
Cumulative incidence of metachronous gastric cancer according to statin use, (**A**) overall, (**B**) until 5 years, and (**C**) until 8 years.

During a median follow-up of 5 years (IQR, 3.5–6.2), metachronous GC developed in 165 (7.6%) patients. Among 165 patients who developed metachronous GC, 13 patients were statin users, and 152 patients were nonusers. Statin users showed a significantly lower cumulative incidence of metachronous GC compared with statin nonusers (Log-rank *p* value = 0.015, Figure 1A). When limited to 5 years, it was not statistically significant (Log-rank *p* value = 0.1, Figure 1B), but, when limited to 8 years, there was a significant difference in incidence of metachronous GC (Log-rank *p* value = 0.018, Figure 1C). In the univariate analysis, statin use was associated with a lower risk of metachronous GC (hazard ratio (HR), 0.50; 95% confidence interval (CI), 0.28–0.85); in contrast, age was associated with the development of metachronous GC (Table 2). In the multivariate analysis, statin use was independently associated with a lower risk of metachronous GC (HR, 0.46; 95% CI, 0.26–0.82). Age was the only factor associated with a higher risk of metachronous GC (HR, 1.03; 95% CI, 1.01–1.05).

### 2.3. Duration and Dose-Response Effects of Statin Use on the Occurrence of Metachronous GC

Table 3 shows the duration and dose-response effects of statin use on the occurrence of metachronous GC. The HRs (95% CIs) of metachronous GC in subjects who used statins for less than 2 years, 2–4 years, and 4 years or more were 0.87 (0.41–1.87), 0.48 (0.15–1.51), and 0.25 (0.08–0.79), respectively, compared to the statin nonusers. In the dose-response effect analysis using cumulative defined daily dose (cDDD), the HRs (95% CIs) of metachronous GC with statin use <500 cDDD, 500–1000 cDDD, and >1000 cDDD were 0.91 (0.46–1.79), 0.64 (0.20–2.01), and 0.08 (0.01–0.58), respectively, compared to those of statin nonusers. In the propensity-score matching cohort (Table 4), a significant inverse dose-response relationship was also observed between statin use and metachronous GC (<500 cDDD: HR 0.63, 95% CI 0.27–1.48; 500–1000 cDDD: HR 0.39, 95% CI 0.09–1.60; >1000 cDDD: HR 0.09, 95% CI 0.01–1.62).

## 3. Discussion

In this cohort study of *H. pylori*-negative patients who underwent endoscopic resection for EGC, we found that statin use was associated with a significant decrease in the risk of GC. Furthermore, significant dose and duration-response effects were observed among patients with a high risk of GC. Since the risk of metachronous GC is high (5-year rate, 3.6% to 16%) [15,16,17,18,19,20,21] in patients who undergo endoscopic resection for EGC, eradication of *H. pylori* could reduce the risk of metachronous GC in patients with *H. pylori* infection [9]. In addition, there is a need to develop novel chemopreventive agents for high-risk patients of GC without *H. pylori* infection.

Previous studies have examined the chemopreventive role of statins in GC; however, the results have been inconsistent [13,22,23,24,25,26]. Randomized controlled trials (RCTs), which included post-hoc analyses for identifying the effect of statins on cardiovascular disease, did not show a chemopreventive effect of statins on the risk of GC, though they found a statistically significant trend (adjusted odds ratio, 0.83; 95% CI, 0.66–1.05) [22,25]. However, the follow-up duration in these studies was not long enough, and the patients were not at a high risk of GC. The chemopreventive effect of statins has been seen in observational studies. A population-based case-control study in Taiwan found that the use of any statin was associated with a lower risk of GC [24]. Another case-control study found that longer the duration of statin use lower was the risk for GC [13]. However, since it was a retrospective case-control study, patients were not routinely screened for GC. The reduced risk of GC observed in statin users might thus be related to the ‘healthy user’ bias [27]. A recent Korean population-based cohort study demonstrated that the use of statin was associated with a reduced risk of metachronous GC in patients who underwent endoscopic resection for EGC [23]. However, the major limitation of the study was the failure to evaluate the status of *H. pylori* infection.

A major limitation of previous studies was the failure to evaluate the status of *H. pylori* infection. A previous meta-analysis of 11 studies found that the use of statin was associated with a reduced risk of GC; however, the included studies did not consider *H. pylori* infection, which is closely related to the risk of GC [25]. A recent study included only patients who were prescribed clarithromycin-based triple therapy for the eradication of *H. pylori*; however, the data of post-treatment *H. pylori* status were unavailable [26]. Since the *H. pylori* eradication rates with clarithromycin triple therapy are reported to be 70–85%, based on the underlying rate of clarithromycin resistance [28], the study might have included patients with persistent *H. pylori* infection. In this study, we evaluated the *H. pylori* status of all patients through a consistent standard method at baseline. Therefore, we included only patients without *H. pylori* infection in the analysis. In addition, a Cox regression analysis adjusted for host factor, detailed tumor characteristics, and important comorbidities, as well as a propensity score matching analysis, were performed to eliminate the potential confounding factors and to minimize the differences between statin users and nonusers. A homogenous cohort, rigorous control of the critical confounders for GC, and a cohort design with annual endoscopic surveillance are the data that provide additional support for the causal relationship between statin use and GC development.

There are several plausible mechanisms to explain the association between statin use and the risk of GC. Large RCTs that analyzed the preventive effects of statins on cardiovascular disease indicated that statins had additional benefits in terms of a decrease in the incidence of colorectal, breast, prostate, and skin cancer [29,30,31,32]. In addition to the HMG-CoA-dependent effect, which is the main mechanism of action of statins, they have important cholesterol/HMG-CoA-independent effects, such as the effect on lymphocyte-function-associated antigen 1 and inhibition of geranylgeranylation, primarily of Rho proteins. These effects that inhibit carcinogenesis involving inflammation, immunomodulation, and angiogenesis are thought to potentially contribute to cancer prevention [33]. In addition, GC is genetically heterogenous disease that progresses through different carcinogenic pathways. Among the genetic changes associated with multi-stage carcinogenesis, C-myc, c-erbB2, K-ras, C-met, TP53, APC, and RUNX3 have been reported [34]. In mice models, inhibition of HMG-CoA reductase by statin inhibits tumor initiation and growth by blocking MYC phosphorylation and activation [35]. In a study using human HGT-1 GC cell line, statin suppressed expression of genes involved in cell division and induced apoptosis of cancer cells overexpressing the MDR-1 gene [36].

Several limitations need to be considered in interpreting these data. First, the histologic examination with special staining methods, such as Giemsa, has been regarded as the gold standard in identifying *H. pylori* infection, with known sensitivity and specificity reaching 95% and 98%, respectively [37]. Although our data might include false negatives, we focused on the effect of statins in the absence of the chemopreventive intervention of *H. pylori* eradication; thus, these would not have affected the results. Second, there might be potential for an inherent selection bias since this was a retrospective observational study. Therefore, a propensity score matching was performed to minimize the differences in the patients’ characteristics between statin users and nonusers. Since this study was conducted in a single-center, surveillance endoscopy was regularly performed at least once a year after endoscopic resection in all patients. There was also no difference in the surveillance interval between statin users and nonusers. Third, although we adjusted for potential confounders, there is still a likelihood of unmeasured residual confounding, such as that caused by smoking, family history of GC, and dietary factors due to the observational nature of our study design. Fourth, a previous study showed that low serum cholesterol levels were an independent risk factor for developing GC [38]; however, our data did not include cholesterol levels. Although the main anticancer effect of statin is explained by cholesterol/HMG-CoA-independent effects rather than the cholesterol-lowering effect, further evaluation is necessary for the effect of changes in cholesterol levels during statin use on the recurrence of GC. Fifth, only Korean patients were included in this study. Koreans have a higher risk of GC than the western populations. Although the results of this study support the effect of statins as chemopreventive agents on GC, external validation in other ethnic groups is necessary to assess the risk-benefit ratio of statins for the prevention of GC.

In conclusion, this study showed that statin use was associated with a significantly lower risk of metachronous GC among high-risk patients without *H. pylori* infection in a dose-and duration-dependent manner. Further prospective studies are needed to confirm this association.

## 4. Materials and Methods

### 4.1. Study Design and Patient Selection

**Figure 2:**
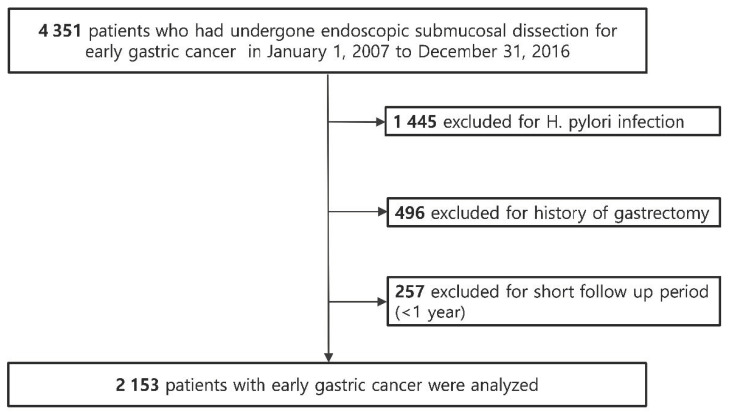
Flowchart of patient enrollment.

We performed a hospital-based retrospective cohort study of patients who underwent endoscopic resection for EGC at the Samsung Medical Center, Seoul, South Korea. Patients were considered eligible if they were ≥20 years old and had undergone endoscopic resection for EGC between January 2007 and December 2016 (*n* = 4351) and followed up until 30 April 2020 (Figure 2). Patients who met any of the following criteria were excluded (*n* = 2505): (i) *H. pylori* infection at the index date (*n* = 1445); (ii) a history of gastrectomy before the index date or during the follow-up period (*n* = 496); and (iii) follow-up duration of less than 1 year (*n* = 257). Finally, a total of 2153 patients without *H. pylori* infection at baseline who underwent endoscopic resection for EGC and were followed up for more than 1 year were included. *H. pylori* infection status was examined routinely during endoscopy at 8 weeks after endoscopic resection for EGC in our center. *H. pylori* infection was evaluated by Giemsa staining of the sample tissues obtained from both the gastric antrum and body. The study protocol was approved by the Institutional Review Board of Samsung Medical Center. As the study used only de-identified data routinely collected during hospital visits, the requirement to obtain informed patient consent was waived.

### 4.2. Study Outcomes

The primary outcome was the incidence of metachronous gastric adenocarcinoma confirmed by endoscopic biopsy. The index date was defined as the date of endoscopic resection for EGC. Metachronous GC was defined as a newly developed GC at least 1 year after the index date. Surveillance endoscopy is routinely performed at least once a year after endoscopic resection for EGC at our institution. The observation period started from the index date and ended when a metachronous GC was diagnosed or at the last clinic visit.

### 4.3. Statin Exposure

We collected the data of statin prescriptions during the follow-up period from our electronic medical record system. We identified the date of prescription, the daily dose, the number of days the drug was given, and the number of pills per statin prescription. Statins included simvastatin, atorvastatin, rosuvastatin, pitavastatin, pravastatin, and fluvastatin.

We defined statin use as at least a 28-day use after the index date. To examine the duration-response relationship, the duration of statin use was divided into four groups: no use, and <2 years, 2–4 years, and ≥4 years of use. To examine the dose-response relationship, the cumulative defined daily dose (cDDD), which is the total of the doses of different statins according to the World Health Organization, was used. The cDDD was calculated as the sum of DDD during the follow-up period. We divided patients into four groups according to the cDDD (no use, and <500, 500–1000, and >1000 mg cDDD).

### 4.4. Covariates

Variables collected were age at index visit, sex, comorbidities (including hypertension, diabetes mellitus, dyslipidemia, cerebrovascular disease, myocardial infarction, heart failure, liver cirrhosis, and chronic kidney disease), and characteristics of index EGC. The characteristics of index EGC included tumor location, maximum size, morphology (elevated, flat, or depressed according to the Paris classification; mixed type), depth of invasion (mucosa or submucosa-invasive), lymphatic or venous invasion, degree of differentiation (based on the World Health Organization’s Classification), and histologic heterogeneity.

### 4.5. Statistical Analysis

Descriptive statistics were used to summarize the patients’ baseline characteristics according to statin use. The cumulative incidences of metachronous GC were evaluated using a Kaplan-Meier curve, and the difference between the curves was tested using the log-rank test. We used a Cox proportional hazard model to estimate the adjusted HR and 95% CI for the development of metachronous GC according to statin use. Age, sex, comorbidities, and tumor characteristics (size, location, morphology, depth of invasion, lymphovascular invasion, histologic differentiation, and histologic heterogeneity) were considered as potential confounders for the association between statin use and metachronous GC. After performing the univariate analysis of each of the potential risk factors for metachronous GC, the variables with a *p* value < 0.1 in the univariate analysis were included in the multivariate analysis. To analyze the dose/duration effect of statin use on the risk of metachronous GC, we used cDDD/duration to assess the risk reduction and compare the risk to that of statin nonusers.

Additionally, propensity score matching was performed using multivariable logistic regression based on the aforementioned covariates to control the selection bias due to different baseline characteristics. Propensity score matching was performed using the nearest-neighbor matching method. The matching balance was evaluated as acceptable if the absolute value of the standardized mean difference was <0.1. After 1:3 propensity score matching, the baseline characteristics between statin users and nonusers were analyzed using a generalized estimating equation (Table 5). A *p-*value < 0.05 was considered statistically significant. All statistical analyses were performed using R version 3.4.3 (R Foundation for Statistical Computing, Vienna, Austria).

## 5. Conclusions

Statin use was associated with a significantly lower risk of metachronous GC among high-risk patients without *H. pylori* infection in a dose- and duration-dependent manner. This finding provides evidence about the additional benefits of statin as chemopreventive agents against metachronous GC among patients who underwent endoscopic resection for EGC. Further prospective studies are required to confirm this association and evaluate risk-benefit in practical use.

## Figures and Tables

**Table 1:** Baseline characteristics of the study cohort.

Variables	Total Patients (N = 2153)	Statin Users (N = 228)	Statin Nonusers (N = 1925)	*p* Value
Age, year	64 (57–71)	66 (60–74)	64 (57–71)	<0.001
Male	1699 (78.9)	195 (85.5)	1504 (78.1)	0.01
Tumor size, mm	12 (8–19)	12 (8–17)	12 (8–20)	0.09
Tumor location				0.22
Lower	1357 (63.0)	73 (62.9)	1224 (63.6)	
Middle	749 (34.8)	42 (36.2)	658 (34.2)	
Upper	47 (2.2)	1 (0.9)	43 (2.2)	
Tumor macroscopic type				0.003
Elevated	270 (12.5)	27 (11.8)	243 (12.6)	
Flat	310 (14.4)	45 (19.7)	265 (13.8)	
Depressed	1009 (46.9)	117 (51.3)	892 (46.3)	
Mixed	564 (26.2)	39 (17.1)	525 (27.3)	
Depth of invasion				0.49
Mucosa (T1a)	1963 (91.2)	212 (93.0)	1751 (91.0)	
Submucosa (T1b)	190 (8.8)	16 (7.0)	174 (9.0)	
Histologic differentiation				049
Differentiated	2148 (99.8)	227 (99.6)	1921 (99.8)	
Undifferentiated	5 (0.2)	1 (0.4)	4 (0.2)	
Histologic heterogeneity				0.40
Absent	2011 (93.4)	210 (92.1)	1801 (93.6)	
Present	142 (6.6)	18 (7.9)	124 (6.4)	
Lymphovascular invasion	43 (2.0)	6 (2.6)	37 (1.9)	0.46
Comorbidities				
Hypertension	378 (17.6)	153 (67.1)	225 (11.7)	<0.001
Diabetes mellitus	240 (11.1)	96 (42.1)	144 (7.5)	<0.001
Myocardial infarction	32 (1.5)	17 (7.5)	15 (0.8)	<0.001
Heart failure	58 (2.7)	29 (12.7)	29 (1.5)	<0.001
Chronic kidney disease	87 (4.0)	40 (17.5)	47 (2.4)	<0.001
Liver cirrhosis	56 (2.6)	8 (3.5)	48 (2.5)	0.36
Cerebrovascular accident	206 (9.6)	87 (38.2)	119 (6.2)	<0.001
Comorbidities (≥2)	380 (17.6)	200 (87.8)	180 (10.4)	<0.001
Aspirin	252 (11.7)	132 (57.8)	120 (6.2)	<0.001
Metformin	199 (9.2)	57 (25.6)	142 (7.4)	<0.001

Values are expressed as median (interquartile range) or frequency (percentage).

**Table 2:** Factors associated with metachronous gastric cancer.

Factors	Univariable Analysis		Multivariable Analysis	
	HR (95% CI)	*p* Value	HR (95% CI)	*p* Value
Age (per year)	1.03 (1.01–1.05)	<0.001	1.03 (1.02–1.05)	<0.001
Male sex	1.20 (0.86–1.79)	0.36		
Tumor size, mm	1.01 (0.99–1.02)	0.07	1.005 (0.99–1.02)	0.505
Tumor location				
Lower	Reference		Reference	
Middle	1.32 (0.96–1.81)	0.08	1.35 (0.99–1.85)	0.056
Upper	1.06 (0.033–3.34)	0.92	0.93 (0.29–2.95)	0.91
Tumor macroscopic type				
Elevated	Reference		Reference	
Flat	0.70 (0.42–1.25)	0.23	0.75 (0.42–1.33)	0.33
Depressed	0.64 (0.40–1.02)	0.06	0.74 (0.47–1.19)	0.22
Mixed	0.91 (0.56–1.48)	0.72	0.98 (0.60–1.58)	0.93
Depth of invasion				
Mucosa (T1a)	Reference			
Submucosa (T1b)	1.00 (0.60–1.69)	0.97		
Histologic differentiation				
Differentiated	Reference			
Undifferentiated	0.05 (0– )	0.66		
Histologic heterogeneity				
Absent	Reference			
Present	1.49 (0.89–2.49)	0.12		
Lymphovascular invasion	1.44 (0.53–3.89)	0.46		
Comorbidities				
Hypertension	0.85 (0.58–1.26)	0.44		
Diabetes mellitus	1.19 (0.76–1.85)	0.38		
Myocadiac infarction	0.67 (0.16–2.73)	0.58		
Heart failure	1.07 (0.47–2.42)	0.87		
Chronic kidney disease	0.97 (0.47–1.98)	0.93		
Liver cirrhosis	1.16 (0.51–2.62)	0.72		
Cerebrovascular disease	1.03 (0.64–1.67)	0.87		
Statin use	0.50 (0.28–0.85)	0.017	0.46 (0.26–0.82)	0.008
Aspirin use	1.21 (0.80–1.82)	0.35		
Metformin use	1.37 (0.85–2.21)	0.19		

HR, hazard ratio; CI, confidence interval.

**Table 3:** Association between duration of use and dose of statins and gastric cancer.

Factors	Multivariable Analysis ^a^	
	HR (95% CI)	*p* Value
Duration		
Non-statin use	Reference	
<2 years	0.87 (0.41–1.87)	0.73
2–4 years	0.48 (0.15–1.51)	0.21
>4 year	0.25 (0.08–0.79)	0.019
cDDD		
Non-statin use	Reference	
<500	0.91 (0.46–1.79)	0.79
500–1000	0.64 (0.20–2.01)	0.44
>1000	0.08 (0.01–0.58)	0.013

^a^ Estimated from Cox proportional hazard models adjusted for variables with a *p* value < 0.10 in the univariate analysis. HR, hazard ratio; CI, confidence interval; cDDD, cumulative defined daily dose.

**Table 4:** Association between duration of use and dose of statins and gastric cancer in the propensity score-matched cohort.

Factors	HR (95% CI)	*p* Value
Statin use	0.34 (0.17–0.71)	0.004
Duration		
Non-statin use	Reference	
<2 years	0.57 (0.23–1.44)	0.235
2–4 years	0.31 (0.07–1.27)	0.104
>4 year	0.18 (0.04–0.75)	0.018
cDDD		
Non-statin use	Reference	
<500	0.63 (0.27–1.48)	0.286
500–1000	0.39 (0.09–1.60)	0.191
>1000	0.09 (0.01–0.62)	0.015

HR, hazard ratio; CI, confidence interval; cDDD, cumulative defined daily dose.

**Table 5:** Baseline characteristics of the propensity score-matched cohort (1:3).

Variables	Statin Users (*n* = 197)	Statin Nonusers (*n* = 425)	*p* Value
Age, year	66 (59–73)	65 (58–72)	0.788
Male	168 (85.3)	339 (79.8)	0.124
Tumor size, mm	12 (8–18)	12 (8–19)	0.671
Tumor location			0.960
Lower	117 (59.4)	248 (58.4)	
Middle	77 (39.1)	171 (40.2)	
Upper	3 (1.5)	6 (1.4)	
Tumor macroscopic type			0.830
Elevated	45 (10.6)	45 (10.6)	
Flat	79 (18.7)	79 (18.6)	
Depressed	212 (49.9)	212 (49.9)	
Mixed	89 (20.9)	89 (20.9)	
Depth of invasion			0.763
Mucosa (T1a)	182 (92.4)	388 (91.3)	
Submucosa (T1b)	15 (7.6)	37 (8.7)	
Histologic differentiation			0.936
Differentiated	196 (99.5)	421 (99.1)	
Undifferentiated	1 (0.5)	4 (0.9)	
Histologic heterogeneity			0.802
Absent	184 (93.4)	393 (92.5)	
Present	13 (6.6)	32 (7.5)	
Lymphovascular invasion	6 (3.0)	10 (2.4)	0.814
Comorbidities			
Hypertension	92 (46.7)	166 (39.1)	0.174
Diabetes mellitus	49 (24.9)	100 (23.5)	0.860
Myocadiac infarction	10 (5.1)	15 (3.5)	0.427
Heart failure	16 (8.1)	26 (6.1)	0.423
Chronic kidney disease	27 (13.7)	38 (8.9)	0.110
Liver cirrhosis	8 (4.1)	16 (3.8)	1.000
Cerebrovascular disease	61 (31.0)	107 (25.2)	0.145

Values are expressed as median (interquartile range) or frequency (percentage).

## Data Availability

The data presented in this study are available on request from the corresponding author. The data are not publicly available due to privacy and ethical restrictions.
